# *Salmonella* Typhimurium outbreak associated with frozen tomato cubes at a restaurant in western Finland, January to February 2021

**DOI:** 10.2807/1560-7917.ES.2022.27.41.2200316

**Published:** 2022-10-13

**Authors:** Sohvi Kääriäinen, Dorothée Obach, Dafni Katerina Paspaliari, Marjut Tofferi, Arto Nieminen, Annika Pihlajasaari, Henry Kuronen, Anni Vainio, Ruska Rimhanen-Finne

**Affiliations:** 1 Finnish Institute for Health and Welfare, Helsinki, Finland; 2 ECDC Fellowship Programme, Field Epidemiology path (EPIET), European Centre for Disease Prevention and Control (ECDC), Solna, Sweden; 3 ECDC Fellowship Programme, Public Health Microbiology path (EUPHEM), European Centre for Disease Prevention and Control (ECDC), Solna, Sweden; 4 Central Ostrobothnia Environmental Health Care, Kokkola, Finland; 5 Central Ostrobothnia Federation on Municipalities for Social and Health Care Services, Kokkola, Finland; 6 Finnish Food Authority, Helsinki, Finland; 7 Finnish Food Authority, Kuopio, Finland

**Keywords:** outbreak, *Salmonella* Typhimurium, tomato, multi-drug resistance, cohort study, whole genome sequencing

## Abstract

Several individuals reported gastrointestinal symptoms following meals consumed in late January 2021 at a restaurant in western Finland. We conducted a retrospective cohort study and defined a case as a person who ate at the lunch restaurant between 27 and 29 January 2021 and had stomach pain, vomiting or diarrhoea and/or a laboratory-confirmed *Salmonella* Typhimurium infection within 2 weeks after the exposure. We collected faecal and food samples for microbiological analysis. *Salmonella* isolates were characterised in detail using whole genome sequencing (WGS) and cluster analysis by core genome multilocus sequence typing (cgMLST). Altogether, 393 meals were sold and 101 people (who ate 142 meals) participated in the cohort study. There were 49 cases; 23 were laboratory-confirmed infections with a multidrug-resistant *S.* Typhimurium. The *S.* Typhimurium isolates from cases and frozen tomato cubes used uncooked in salads were closely related and clustered together in cgMLST comparison. These salads were consumed by 76% of the cases. Based on the cgMLST clustering, they were the suggested source of the outbreak. Statistical association was not significant between eating the salads and being a case. Following the outbreak investigation, the producer decided to recommend cooking of their frozen tomato products before consumption.

**Key public health message**

**What did you want to address in this study?**

Our aim was to identify the source of a gastrointestinal outbreak caused by multidrug-resistant *Salmonella* in order to prevent the expansion of the outbreak and similar outbreaks in the future.  **What have we learnt from this study?**

The source of the outbreak was a frozen tomato product, which to our knowledge has not been reported before. This outbreak highlights the importance of cooking recommendation for food products to prevent outbreaks. Two asymptomatic staff members were found to be positive for *Salmonella*, which encourages the testing of asymptomatic food handlers during gastrointestinal outbreaks. **What are the implications of your findings for public health?**

No national recommendations for cooking frozen tomato products exist in Finland. Prompted by the findings of the outbreak investigation, the producer decided to recommend the cooking of their frozen tomato products before consumption. Outbreaks of multidrug-resistant strains originating from contaminated food can contribute to the introduction of antibiotic resistant strains to Finland.

## Background

*Salmonella* infections are the second most commonly reported cause of gastroenteritis and important causes of food-borne outbreaks in the European Union and European Economic Area (EU/EEA) [1]. The incidence of *Salmonella* infections was 13.7 per 100,000 in EU/EEA in 2020. *Salmonella* infections are notifiable in Finland, and notifications are sent by microbiological laboratories to the National Infectious Diseases Register (NIDR). The average number of cases each year was 1,500 in between 2014 and 2019 [2]. The majority of these infections were acquired abroad [3]. Probably as a consequence of the pandemic restrictions on foreign travel, the annual number of salmonellosis cases in Finland decreased to 516 in 2020, an incidence of 9.3 per 100,000 population, of which 38% were acquired abroad. There were 37 different serotypes causing domestic infections in 2020. The most common serotypes among the domestic strains were *Salmonella* Typhimurium, including monophasic variants, *S*. Saintpaul and *S.* Enteritidis, corresponding to 64% of all domestic infections [4].

Antimicrobial resistance is rare in domestic *Salmonella* isolates in Finland. In the year 2020, only two among the 48 non-monophasic *S.* Typhimurium strains were multidrug-resistant, and 22 of 24 *S.* Enteritidis isolates were sensitive to all tested antimicrobials [4]. Among monophasic S. Typhimurium isolates, antimicrobial resistance was common, 25 of 27 isolates were multidrug-resistant in 2020.

## Outbreak detection

On 2 February 2021, a local environmental health authority in western Finland notified the National Registry for Food and Waterborne Outbreaks about six cases of *Salmonella* infection suspected to be related to a local lunch restaurant in a region where there had been between one and three salmonellosis cases per month. More cases were soon discovered that were linked to food eaten at the same restaurant on the days 27–29 January 2021, and the Finnish Institute for Health and Welfare (THL) was informed of 44 outbreak-related cases. The estimated number of potentially exposed persons was 400. THL and the Finnish Food Authority joined the local outbreak investigation team to support and coordinate the epidemiological and microbiological investigations.

We report here a food-borne outbreak caused by a non-monophasic multidrug-resistant *S.* Typhimurium strain that was not detected in Finland before.

## Methods

### Epidemiological investigation

We conducted a retrospective cohort study to identify the source of the outbreak. We published a press release on 5 February 2021 in a local newspaper and on social media to inform the public about the current outbreak and to reach people who had eaten at the restaurant on any day between 27 and 29 January 2021. We asked exposed persons to fill in an electronic notification form that asked questions about exposure and possible symptoms. Local healthcare units were informed about the outbreak in order to find cases. Both symptomatic and asymptomatic exposed people were interviewed by telephone by the local environmental health authority using a specific questionnaire for each day of the exposure period. In the questionnaire, we asked for the time of the exposure and exposure to each food item served that day. Also, we asked about the appearance of symptoms (diarrhoea, vomiting, stomach pain, nausea, fever (≥ 38 °C), chills, headache) and the timing of the first symptom.

Finnish clinical microbiology laboratories notify all *Salmonella* findings to NIDR. Notifications include personal data, date and type of the specimen, laboratory method and preceding travel history. Isolates of domestic and of all invasive non-typhoidal *Salmonella* infections are sent to the national reference laboratory at THL for serotyping. All domestic isolates are sequenced. The infectious diseases unit in the healthcare district where outbreak took place was closely involved in the outbreak investigation and monitored the number and results of *Salmonella* samples requested from the public primary care and secondary care in that region.

#### Exposure definition

We defined exposure as having eaten lunch in the lunch restaurant on any of the days from 27 to 29 January 2021.

#### Case definition

We defined a case as a person who ate at the restaurant between 27 and 29 January 2021, had a laboratory-confirmed *Salmonella* Typhimurium infection by PCR or culture, and/or at least one of the following symptoms: stomach pain, vomiting and diarrhoea within 2 weeks of having had a meal at the restaurant.

#### Statistical methods

We calculated food-specific attack rates (AR), relative risks (RR) and 95% confidence intervals (CI) for single and pooled exposures. We pooled the food items that were served on multiple days when they came from the same batch or arrived on the same day at the restaurant or if the same food item or meal was re-used in the following days. Stratified analyses were performed for the food items that were hypothesised to be associated with *Salmonella* infection. We analysed all side salads (lettuce, tomato, cucumber, green beans, tomato salad, rice salad and lingonberry) grouped as one exposure and did a pooled analysis for the exposure period. As there were no unexposed cases on 28 and 29 January 2021 in this analysis, we added one person to each group (exposed case, unexposed case, exposed non-case, unexposed non-case) (n = 105). We also did an analysis where symptomatic persons with *Salmonella*-negative stool samples were classified as non-cases. For categorical variables proportions were compared using chi-square test. The level of statistical significance was set to 0.05. The analyses were performed using Stata 16.1.

### Environmental investigation

The local environmental health authority inspected the restaurant’s premises on 2 February 2021. They collected the menu lists and went through the food preparation process. Most food items were bought from one wholesaler. A nearby grocery store was occasionally used for purchases.

The restaurant was only open on Monday to Friday during lunch hours. The lunch buffet selection was mostly different each day (Table 1). In addition, people could order meals from an à-la-carte menu or buy items to supplement the lunch buffet. Salads at the buffet were included when ordering meals from the à-la-carte menu. The restaurant was using uncooked frozen tomato cubes for salads served in the lunch buffet each day on 27–29 January 2021. The restaurant sold overall 152 portions of food on 27 January, 127 portions on 28 January and 114 portions on 29 January, totalling 393 portions during the studied period.

**Table 1:** Food items served in the restaurant and samples taken from the food items, *Salmonella* Typhimurium outbreak, western Finland, January–February 2021 (n = 20)

Food item	Day(s) served	Style of serving	Sample taken	Sample date	Batch number	Number of samples	Laboratory method^a^
Butter chicken	27	Buffet	No	NA			
Rice	27	Buffet	No	NA			
Tomato salad^b^	27–29	Buffet	Yes	8 Feb	08.2022	1	BACGene qPCR
				8 Feb	10.2022	1	BACGene qPCR
				10 Feb	10.2022	1	BACGene qPCR
				11 Feb	10.2022	4	BACGene qPCR
				15 Feb	07.2022	1	In-house RT-PCR (method TL25)
				15 Feb	08.2022	1	In-house RT-PCR (method TL 25)
				15 Feb	08.2022	2	Vidas SPT [5] and enrichment culturing ISO 6579–1:2017 [6]
Green beans	27–29	Buffet, à la carte	Yes	8 Feb	NA	2	Enrichment culturing NMKL 187;2016 [7]
Lettuce	27–29	Buffet	Yes	4 Feb	NA	4	BACGene qPCR
Yogurt sauce	27	Buffet	No	NA			
Chickpea stew	27	Buffet	No	NA			
Naan bread	27	Buffet	No	NA			
Beef red wine	28	Buffet	No	NA			
Butter potato	28	Buffet	No	NA			
Cabbage stew	28	Buffet	No	NA			
Cucumber	28–29	Buffet	No	NA			
Tomato (fresh)	28–29	Buffet	No	NA			
Lingonberry	28	Buffet	No	NA			
Bread	28–29	Buffet	No	NA			
Spread	28–29	Buffet	No	NA			
Pulled pork	29	Buffet	No	NA			
Baked potato	29	Buffet	No	NA			
Salmon mousse	29	Buffet	No	NA			
Cooked vegetables	29	Buffet	No	NA			
Chocolate cake	27, 29	À la carte	No	NA			
Sweet potato	28–29	À la carte	No	NA			
Cream	28–29	À la carte	No	NA			
Steak	28	À la carte	No	NA			
Chips	28	À la carte	No	NA			
Beetroot	28	À la carte	No	NA			
Vegetable stew	28–29	À la carte	No	NA			
Canned peach	27–29	Buffet	No	NA			
Pumpkin seeds	27–29	Buffet	No	NA			
Garam masala spice	27	Buffet	Yes	2 Feb	NA	1	BACGene qPCR
Eggs	NA	NA	Yes	8 Feb	NA	1	BACGene qPCR
Peanuts	27–29	Buffet	Yes	8 Feb	NA	1	BACGene qPCR

NA: not available.

^a^ Samples were analysed in four different local laboratories. For samples positive with PCR or VIDAS SPT [5], results were cultured to confirm the finding.

^b^ The variable ‘Tomato salad’ includes tomato salad and rice salad, which contained the frozen tomato cube product.

There were four staff members in the restaurant, and they were all asymptomatic, as reported on 10 February 2021. They had been eating the same food that was sold in the restaurant during the days 27–29 January 2021. Stool samples from the staff members were requested by the occupational healthcare.

Two more visits were made to the restaurant during the outbreak investigation on 4 and 8 February 2021 to collect more food and surface samples.

### Microbiological investigation

#### Clinical laboratory testing for human samples

Most of the outbreak-related laboratory tests were carried out by the public healthcare on stool samples. Twelve cases were tested with a PCR test that included *Shigella*/enteroinvasive *Escherichia coli*, *Campylobacter jejuni*/*coli*, *Vibrio vulnificus*/*parahaemolyticus*/*cholerae*, enterotoxigenic *E. coli*, enterohaemorrhagic *E. coli*, *Salmonella*, *Plesiomonas shigelloides* and *Yersinia enterocolitica.* The analysis was continued with culture if the PCR test was positive. Five cases were tested with this PCR test and with bacterial culture for *Staphylococcus aureus*, *Clostridium perfringens* and *Bacillus cereus*. One additional case was tested by PCR for bacterial pathogens and norovirus. Finally, after *Salmonella* was revealed as the causative agent, five further cases were tested only for *Salmonella* by bacterial culture.

In the clinical laboratories, samples positive for *Salmonella* in the PCR test were cultured, and isolates from the culture-positive samples were sent to the THL reference laboratory.

#### Samples of food items and surface samples

Samples of food items (Garam masala spice in an opened package, 300 g of chopped lettuce collected to a clean container, uncut lettuce, two randomly sampled eggs and peanuts in an opened bag) were collected from the restaurant between 2 and 8 February 2021 (Table 1). There were no samples of the salads containing frozen tomato cubes left over for analysis, but two samples of frozen tomato cubes (batches 08.2022 and 10.2022) were collected from unopened packages (2.5 kg each) from the wholesaler on 8 February 2021 and one sample on 10 February 2021 (batch 10.2022) for microbiological analysis (Table 1). 

The wholesaler also took eight additional samples from the frozen tomato cubes at three different locations of the company in Finland from three batches. A sample of green beans (two bags of 2.5 kg) was collected by environmental health from the same wholesale business in another town on 8 February 2021.

Samples of food items were analysed in four different local laboratories by BACGene qPCR, in-house RT-PCR, VIDAS SPT [5] or by enrichment culturing methods ISO 6579–1:2017 [6] or the Nordic Committee on Food Analysis (NMKL) reference culture method 187:2016 [7] (Table 1). The samples with positive PCR or VIDAS SPT results were cultured to confirm the finding.

During a visit to the restaurant on 2 February 2021, 13 surface samples (toilet, bathroom samples, worktop with two taps, oven, refrigerator, kitchen sink, two cutting boards, shelving units, cash register, worktop for microwave, worktop for bread and a pan) were collected for microbiological analyses for *Salmonella*. Ten more surface samples (food serving desk, hand basin, worktop for lunch and tap, tea towel and gloves, worktop for salads, worktop for pizza, staff bathroom, staffroom and changing room, cleaning equipment and cold storage) were collected on 8 February 2021. Surface samples were analysed by BACGene qPCR.

#### Microbiological investigation in the reference laboratories

In this study, all *Salmonella* Typhimurium isolates from patient samples were sent to THL for serotyping, antimicrobial susceptibility testing and whole genome sequencing (WGS). Isolates were serotyped by slide agglutination [8], and WGS was done using the Nextera XT DNA Library Preparation Kit and MiSeq sequencer (Illumina Inc). Multilocus sequence typing (MLST) and cluster analysis (cgMLST) from sequence data were done using Ridom SeqSphere+ [9-11]. Briefly, we used the Ridom SeqSphere+ software’s Target Definer tool with default parameters to identify 3,696 targets defined for cgMLST shared by the Salmonella Reference Genome NC_011294.1 (*Salmonella enterica* subsp. *enterica* serovar Enteritidis str. P125109 complete genome) and 48 additional complete query genomes obtained from GenBank. All samples were additionally tested for susceptibility to ampicillin, cefotaxime, chloramphenicol, gentamicin, mecillinam, meropenem, nalixidic acid, pefloxacin, streptomycin, sulfonamides, tetracycline and trimethoprim, through disk diffusion, as described by Huusko et al. [12].

The isolate from the tomato cubes was sent for typing by slide agglutination [13] to the laboratory in the Finnish Food Authority and from there to THL for WGS. The WGS for the isolate was done using the Nextera XT DNA Library Preparation Kit and MiSeq sequencer at THL.

## Results

### Epidemiological investigation

#### Descriptive epidemiology

In total, 101 persons, who ate 142 meals over the exposure period, participated in the retrospective cohort study. Of those, 49 persons (49%) fulfilled the case definition. 61% of the participants were male, and the median age of the cohort was 39 years (range: 16–77). Cases and non-cases did not differ in sex or age distribution (Table 2), the majority were in the age category 26 to 45 years.

**Table 2:** Sex and age distribution of cases and non-cases with attack rate, relative risks and 95% confidence intervals, *Salmonella* Typhimurium outbreak, western Finland, January–February 2021 (n = 101)

	Cases (n)	Non-cases (n)	AR (%)	RR (95% CI)	p value
All	49	52	48.5	NA	
Sex					
Female	19	20	48.7	Reference	
Male	30	32	48.4	1.0 (0.66–1.50)	0.974
Age categories (years)^a,b^					
≤ 25	3	2	60.0	1.31 (0.61–2.78)	0.542
26–35	10	11	47.6	1.02 (0.60–1.73)	0.933
36–45	17	16	51.5	1.18 (0.74–1.89)	0.480
46–55	6	6	50.0	1.08 (0.58–2.01)	0.811
> 55	1	7	12.5	0.25 (0.04–1.56)	0.040

AR: attack rate; CI: confidence interval; NA: not applicable; RR: relative risk.

^a^ No information for 22 people.

^b^ Each age category was compared with the rest of the cohort.

**Figure 1:**
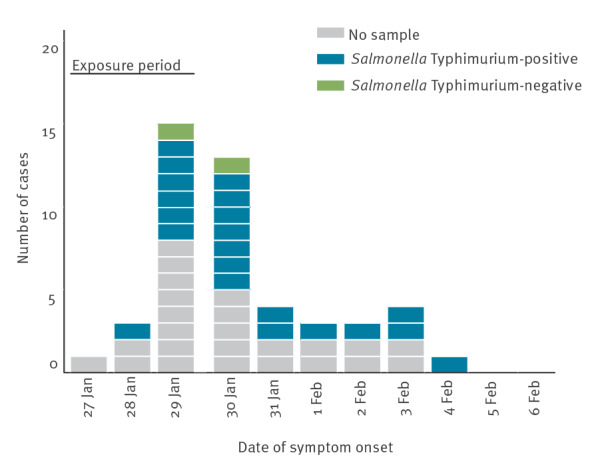
Cases by date of symptom onset, *Salmonella* Typhimurium outbreak, western Finland, January–February 2021 (n = 47)

**Figure 2:**
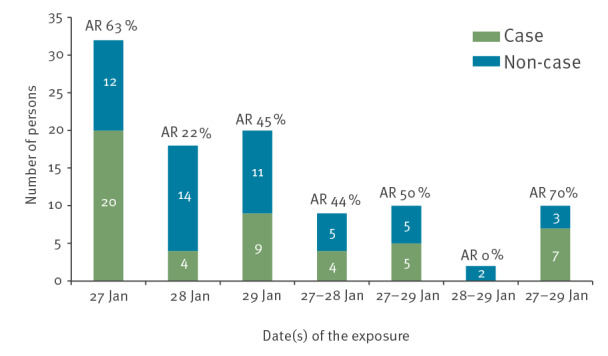
Number of cases and non-cases by date of the exposure, *Salmonella* Typhimurium outbreak, western Finland, January–February 2021 (n =101)

Based on the symptom onset, the peak in the number of cases (57%) was on 29 and 30 January 2021, and the cases decreased from 31 January 2021 onwards, suggesting a point source (Figure 1). The exposure took place in a single restaurant over 3 days. The AR was highest for people who ate at the restaurant on 27 January 2021, and 36 of the 49 cases had lunch on that day (Figure 2). Diarrhoea and stomach pain were the most common symptoms among the cases, 39 and 42 of 49, respectively (Table 3). Two cases were hospitalised, one of whom had a *Salmonella*-positive blood culture. There were no deaths.

Missing date for two cases. Positivity as per either PCR or culture.

AR: attack rate.

**Table 3:** Frequency of symptoms^a^ in cases and non-cases in the *Salmonella* Typhimurium outbreak in western Finland, January–February 2021 (n = 101)

Symptoms	Cases (n = 49)	Non-cases (n = 52)
Stomach pain	42	0
Diarrhoea	39	0
Headache	28	2
Nausea	24	1
Fever (≥ 38 °C)	17	2
Chills	18	0
Vomiting	3	0

^a^ A person could select several answers.

Two persons who fulfilled the case definition based on the symptom criteria had a negative stool sample for *Salmonella*. Stool samples for these two cases were taken 8 and 11 days after symptom onset.

#### Clinical laboratory testing of human samples

*Salmonella* Typhimurium was isolated from 23 stool samples from the cases that participated in the cohort study. In addition, one case was identified after the study at the THL laboratory by WGS. The case lived in a neighbouring region and the epidemiological link to the restaurant was confirmed by telephone after the WGS result. *Salmonella* was the only positive finding in the analysis of outbreak-related stool samples. One person had *Salmonella* both in the stool sample and blood culture.

The four staff members in the restaurant participated in the cohort study, all were asymptomatic and gave stool samples. Two were positive for *Salmonella* Typhimurium and were counted as cases in the outbreak investigation.

In the healthcare district where the outbreak took place, the number of faecal samples sent for bacterial microbiological analysis was 65–88 per month during the 3 months before the outbreak. Testing increased to 101 per month after the start of the outbreak. In February 2021, 19 salmonellosis cases were reported. During the whole year 2021, 30 *Salmonella* infections were reported in the healthcare district, of which 23 were linked to this outbreak. The incidence in 2021 was 2.6-fold higher than in the years 2018 to 2020 (39/100,000 vs 14–16/100,000 population).

#### Analytical epidemiology

None of the food items, except the chocolate cake, were associated with being a case in the pooled analysis (Table 4). The chocolate cake, despite having a significant association, was only eaten by three people and was therefore unlikely to explain the outbreak. An association of borderline significance was found between lettuce and being a case, with a risk ratio of 4.5 (p = 0.026; 95% CI: 0.70–28.90) (Table 3).

**Table 4:** Food-specific attack rates, relative risks, 95% confidence intervals and percentage of cases exposed in pooled analysis, *Salmonella* Typhimurium outbreak, western Finland, January–February 2021 (n = 49)

Exposure	Food eaten			Food not eaten			Relative risk		p value	% of cases exposed
	Total	Cases	AR	Total	Cases	AR	RR	95% CI		
Chocolate cake	3	3	100.0	47	16	34.0	2.94	1.97–4.37	0.022	15.8
Lettuce	86	43	50.0	9	1	11.1	4.50	0.70–28.90	0.026	97.7
Baked potato	34	19	55.9	7	1	14.3	3.91	0.62–24.61	0.045	95.0
Lingonberry	19	10	52.6	17	4	23.5	2.24	0.86–5.83	0.074	71.4
Tomato salad	66	37	56.1	16	5	31.2	1.79	0.84–3.83	0.075	88.1
Beef red wine	34	15	44.1	4	0	0.0	∞	NA	0.088	100.0
Pulled pork	39	20	51.3	2	0	0.0	∞	NA	0.157	100.0
Chips	3	0	0.0	34	13	38.2	0.00	NA	0.184	0.0
Steak	3	0	0.0	34	13	38.2	0.00	NA	0.184	0.0
Naan bread	43	28	65.1	16	8	50.0	1.30	0.76–2.23	0.290	77.8
Chickpea stew	20	9	45.0	31	18	58.1	0.77	0.44–1.37	0.361	33.3
Cabbage stew	20	9	45.0	16	5	31.2	1.44	0.60–3.45	0.400	64.3
Yogurt sauce	39	23	59.0	11	5	45.4	1.30	0.65–2.61	0.425	82.1
Cooked vegetables	27	13	48.1	9	3	33.3	1.44	0.53–3.94	0.439	81.2
Salmon mousse	28	15	53.6	12	5	41.7	1.29	0.61–2.73	0.490	75.0
Butter potato	31	13	41.9	7	2	28.6	1.47	0.42–5.08	0.514	86.7
Cucumber	57	21	36.8	4	2	50.0	0.74	0.26–2.08	0.600	91.3
Green beans	19	12	63.2	39	22	56.4	1.12	0.72–1.74	0.624	35.3
Rice	58	34	58.6	2	1	50.0	1.17	0.29–4.77	0.808	97.1
Spread	24	9	37.5	32	13	40.6	0.92	0.47–1.79	0.813	40.9
Bread	25	9	36.0	32	12	37.5	0.96	0.48–1.91	0.907	42.9
Tomato (fresh)	51	20	39.2	10	4	40.0	0.98	0.43–2.25	0.963	83.3
Cream	2	0	0.0	0	0	NA	NA			
Butter chicken	61	36	59.0	0	0	NA	NA			
Beetroot	3	0	0.0	1	0	0.0	NA			
Sweet potato	2	0	0.0	0	0	NA	NA			
Vegetable stew	2	0	0.0	0	0	NA	NA			

AR: attack rate; CI: confidence interval; NA: not applicable; RR: relative risk.

The results were the same when the two cases with a negative stool sample were classified as non-cases (Supplementary Table S1 shows the results of this sensitivity analysis). We also analysed all side salads as a grouped variable for each day and in a pooled analysis. The AR was 50 of 101 (49.5%) among the exposed cases and one of four among the unexposed cases, resulting in an RR of 1.98 (95% CI: 0.36–10.93; p = 0.336).

### Environmental investigation

During the inspection visit to the restaurant on 2 February 2021, it was noted that there was ongoing renovation on the premises with some ensuing disorder. The temperature logs for refrigerators and other cooling devices were checked and found to be in accordance with the legislation. The meals served in the buffet were cooked in the morning on the day of serving them, and they were served next to each other on the buffet. Cold and hot plates were used for the serving of cold and hot meals, respectively in the lunch buffet. For hot meals, heat lamps were also used. The serving temperatures fulfilled the requirements of the legislation.

The company purchases food products from a local wholesaler three to four times a week. Food items were transported in coolers, and the transport time was short, i.e. about 10 min. Meat products were mainly Finnish products, whereas frozen vegetables were imported or within intra-EU trade. Frozen tomato cubes were used uncooked for a tomato salad with onion and cucumber which was served on 27 and 29 January 2021. This salad was mixed with rice and served as a rice salad on 28 January 2021.

#### Microbiological investigation of food and surface samples

One of two frozen tomato cube samples taken on 8 February 2021 (batches 08.2022 and 10.2022) from the local wholesaler (unopened package) was positive for *Salmonella* in PCR and culture. All other samples of food items collected from the restaurant and the local wholesaler on 2 and 8 February 2021 were *Salmonella-*negative as well as the one sample from frozen tomato cubes collected from the wholesaler on 10 February 2021 (batch 10.2022). Eight additional tomato cube samples from wholesalers from different towns, which included samples from the batches 07.2022, 08.2022 and 10.2022, were all *Salmonella-*negative, as well as the surface samples from the restaurant.

#### Microbiological investigation in the reference laboratories

All the isolates were identified as *Salmonella* Typhimurium with antigenic structure 4,12:i:1,2. Based on WGS data, all isolates were typed as MLST sequence type (ST) 19. In cgMLST analysis of 3,235 target alleles (after excluding 270 missing alleles), all isolates from the human specimens as well as the one from the tomato cubes were found to be closely related, with a maximum of two allelic differences.

Antimicrobial susceptibility testing of all isolates revealed that the outbreak strain was multidrug-resistant, exhibiting resistance to ampicillin, chloramphenicol, streptomycin, sulfonamides, tetracycline, pefloxacin and nalidixic acid. The strain was susceptible to trimethoprim, gentamicin, cefotaxime, mecillinam and meropenem.

## Outbreak control measures

Local healthcare workers were informed by the infectious diseases unit about salmonellosis cases related to the restaurant on 4 February 2021 in order to identify cases and increase the testing of patients with gastroenteritis symptoms. Two press releases were published, on 5 and 8 February 2021, to inform the public about the outbreak and invite exposed people to contact the local environmental health authority. During the outbreak investigation, two further press releases (11 and 19 February 2021) were published to communicate the results of the investigation.

The local environmental health authority advised the restaurant to intensify cleaning at the beginning of the outbreak and repeatedly after that. It was requested that fabric towels in the staff bathroom should be replaced with disposable ones. The restaurant was also advised to freeze food samples in the future.

The product of frozen tomato cubes came to Finland as intra-EU trade and the wholesaler company acted as the importer. The product was not sold directly to consumers. The wholesale business issued an initial internal withdrawal of the two batches of the product on 12 February when the PCR test of the frozen tomato cube product came back positive for *Salmonella* and informed the customers who had bought the product. After confirmation of *Salmonella* Typhimurium by culture, the withdrawal was extended to all batches of the product on 17 February 2021. Information was shared also on the webpage of the wholesaler. The notification was sent to the Finnish Food Authority, local environmental health and food authorities in the municipalities of the wholesaler and the restaurant where the outbreak took place. The wholesaler ceased selling the product on 10 February until the producer put in place the new labelling.

A Rapid Alert System for Food and Feed (RASFF) notification was not done as there was uncertainty about the batch number of the contaminated product. Samples from two different batches (one each) of frozen tomato cubes were sent on the same day from the wholesaler and one of them was positive for *S.* Typhimurium. However, we could not say from which one of the two batches the positive sample was taken. Both batches were included in the withdrawal in Finland.

The wholesaler informed the producer about the *Salmonella* finding in the product, after which the producer added a label to the package that the product of frozen tomato cubes must be cooked before consumption.

## Discussion

*Salmonella* Typhimurium was found both in the patient samples and in a frozen product of tomato cubes that was used uncooked in two different salads. These salads were served on all of the days of the outbreak period. The *S.* Typhimurium isolates from the cases’ samples were very closely related to each other and to the one isolate from the frozen tomato cubes. This suggests that the frozen tomato product was the source of the outbreak.

Even though *S.* Typhimurium was found in the tomato cubes and stool samples, no significant statistical association was found between the consumption of salads that included the raw food item and being a case. However, the majority of the cases reported an exposure to the salads supporting the findings of the microbiological analysis.

Increasing rates of antimicrobial resistance in *S.* Typhimurium have been reported globally, especially for monophasic *S.* Typhimurium [14,15]. Fluoroquinolone resistance has mainly remained low for *Salmonella*, but there are remarkable differences between serovars and countries [16,17]. Fluoroquinolone resistance among *Salmonella* species has been identified by the World Health Organization as a high-priority concern [18]. The outbreak strain was resistant to multiple antibiotics, including fluoroquinolones. Outbreaks of multidrug-resistant strains originating from contaminated food items outside Finland can contribute to the introduction of antibiotic resistant strains.

*Salmonella* was found in one package of frozen tomato cubes, which came from the local wholesaler. Other packages of the same product from the same and different batches were negative for *S.* Typhimurium. We could not trace the origin of the contamination. However, fresh products have increasingly been detected as sources of *Salmonella* outbreaks [19,20], and contamination is possible at the production sites, both before and after harvest [21]. Tomatoes are no exception; especially in the United States, *Salmonella* outbreaks related to tomatoes have often been reported [22].

*Salmonella* can survive in soil, which it may have entered through different routes such as manure or irrigation water, and contaminate the tomato plant [23,24]. However, contamination of tomato fruit was rare in an experimental setting in natural conditions even though *Salmonella* was isolated from irrigation water, organic fertilisers, soil and the leaves of tomatoes on a field [25]. The rate of contamination may be reduced by agricultural practises, such as using mulch. The contamination level is shown to be different depending on the tomato cultivar and on the serovar and strain of *Salmonella* [24,26]. Therefore, selecting tomato cultivars that are less prone to *Salmonella* and favouring protective practices in agriculture could prevent outbreaks in future.

Tomatoes have rarely been implicated as the source of *Salmonella* outbreaks in Europe [20,27,28]. We were able to identify the source of the outbreak in microbiological analysis, which showed a multi-resistant *Salmonella* strain. Furthermore, this outbreak was caused by a frozen tomato product. No national recommendations for cooking frozen tomato products exist in Finland, whereas there is a recommendation to cook imported frozen berries because of the risk of gastrointestinal viruses, and also a recommendation for certain medical risk groups to cook frozen vegetables because of the risk of *Listeria*. Following our outbreak investigation, the producer decided to recommend cooking of their frozen tomato products before consumption.

There are some limitations in this study. The majority of the food items were served as a self-serve buffet, which may have affected how well restaurant customers remember the food items they ate. In addition, buffet-style serving makes it difficult to assess the size of the consumed portion. People ordering à-la-carte meals had the possibility to eat salads from the lunch buffet, but these were only a few. Some other food items such as the chocolate cake were not included in the lunch buffet but could be bought separately; these were consumed by very few people.

We found a borderline association between the consumption of lettuce and being a case. This could be explained by possible cross-contamination because 84% of the cases exposed to lettuce and tomato cubes ate both of these items. Also, the same utensils might have been used to take lettuce and salad with tomato cubes, or the containers for the food items may have been placed next to each other. Furthermore, using press releases to reach exposed people could have caused selection bias, i.e. those who had symptoms may have been more eager to participate in the study. Also, symptomatic persons may have remembered better the dishes they ate before getting ill, causing a recall bias.

Lastly, we were not able to reach all of the exposed people, which probably decreased the power in the statistical analysis of the cohort study. Altogether, 393 portions were sold at the restaurant during the outbreak period, while 101 persons answered the questionnaire. They had consumed 36% of the sold portions as some of them visited the restaurant more than once during the 3 days. We used a retrospective cohort study since a group of people visiting the same restaurant could be defined. We also analysed the data using case–control setting, which provided similar results (Supplementary Table S2).

## Conclusions

We were able to identify a frozen product of tomato cubes as the source of the outbreak by sequencing isolates from patient samples and a food sample. This highlights the importance of proper labelling of food products to prevent outbreaks. Two asymptomatic staff members were found to be positive for *Salmonella*, which encourages the testing of asymptomatic food handlers during gastrointestinal outbreaks.

## Supplementary Data

203000 Supplement
