# Supplementary material for: Salmonella Typhimurium outbreak associated with frozen tomato cubes at a restaurant in western Finland, January to February 2021
Source: Euro Surveill. 2022 Oct 13;27(41):2200316. doi: 10.2807/1560-7917.ES.2022.27.41.2200316 (PMC9562807; doi:10.2807/1560-7917.ES.2022.27.41.2200316)
Supplement: Supplement [file 22-00316_KAARIAINEN_Supplement.pdf]

This supplementary material is hosted by *Eurosurveillance* as supporting information alongside the article '*Salmonella* Typhimurium outbreak associated with frozen tomato cubes at a restaurant in western Finland, January to February 2021', on behalf of the authors, who remain responsible for the accuracy and appropriateness of the content. The same standards for ethics, copyright, attributions and permissions as for the article apply. Supplements are not edited by *Eurosurveillance* and the journal is not responsible for the maintenance of any links or email addresses provided therein.

Supplementary Table S1. Food-specific attack rates, relative risks, 95% confidence intervals and percentage of cases exposed in pooled analysis, *Salmonella* Typhimurium outbreak, western Finland, January–February 2021. The two *Salmonella* negative cases are not counted as cases in this sensitivity analysis.

Exposure	Food eaten			Food not eaten			Relative risk		p value
	Total	Cases	AR	Total	Cases	AR	RR	95% CI	
Chocolate cake	3	3	100.0	47	16	34.0	2.94	[1.97–4.37]	0.022
Lettuce	86	41	47.7	9	1	11.1	4.29	[0.67–27.59]	0.036
Baked potato	34	18	52.9	7	1	14.3	3.71	[0.59–23.38]	0.062
Lingonberry	19	10	52.6	17	4	23.5	2.24	[0.86–5.83]	0.074
Tomato salad	66	35	53.0	16	5	31.2	1.70	[0.79–3.63]	0.118
Beef red wine	34	15	44.1	4	0	0.0	∞	NA	0.088
Pulled pork	39	19	48.7	2	0	0.0	∞	NA	0.178
Chips	3	0	0.0	34	13	38.2	0.00	NA	0.184
Steak	3	0	0.0	34	13	38.2	0.00	NA	0.184
Naan bread	43	27	62.8	16	7	43.7	1.44	[0.79–2.62]	0.188
Chickpea stew	20	9	45.0	31	16	51.6	0.87	[0.48–1.58]	0.645
Cabbage stew	20	9	45.0	16	5	31.2	1.44	[0.60–3.45]	0.400
Yogurt sauce	39	22	56.4	11	5	45.4	1.24	[0.61–2.51]	0.520
Cooked vegetables	27	13	48.1	9	3	33.3	1.44	[0.53–3.94]	0.439
Salmon mousse	28	14	50.0	12	5	41.7	1.20	[0.56–2.58]	0.629

Exposure	Food eaten			Food not eaten			Relative risk		p value
	Total	Cases	AR	Total	Cases	AR	RR	95% CI	
Butter potato	31	13	41.9	7	2	28.6	1.47	[0.42–5.08]	0.514
Cucumber	57	20	35.1	4	2	50.0	0.70	[0.25–1.99]	0.548
Green beans	19	12	63.2	39	21	53.8	1.17	[0.75–1.84]	0.502
Rice	58	32	55.2	2	1	50.0	1.10	[0.27–4.50]	0.885
Spread	24	9	37.5	32	12	37.5	1.00	[0.50–1.98]	1.000
Bread	25	9	36.0	32	11	34.4	1.05	[0.52–2.13]	0.898
Tomato (fresh)	51	19	37.2	10	4	40.0	0.93	[0.40–2.15]	0.870
Cream	2	0	0.0	0	0	NA	NA		
Butter chicken	61	34	55.7	0	0	NA	NA		
Beetroot	3	0	0.0	1	0	0.0	NA		
Sweet potato	2	0	0.0	0	0	NA	NA		
Vegetable stew	2	0	0.0	0	0	NA	NA		

AR: attack rate; CI: confidence interval; NA: not applicable; RR: relative risk

Supplementary Table S2. Food-specific attack rates, odds ratios, 95% confidence intervals and percentage of cases and controls exposed in pooled analysis, *Salmonella* Typhimurium outbreak, western Finland, January–February 2021 using a case-control study design.

Exposure	Cases			Controls			Odds ratio		p value
	Total	Exposed	AR	Total	Exposed	AR	OR	95% CI	
Chocolate cake	19	3	15.8	31	0	0	NA	NA	0.022
Lettuce	44	43	97.7	51	43	84.3	8.00	[0.98–363.00]	0.026
Baked potato	20	19	95.0	21	15	71.4	7.60	[0.76–367.51]	0.045
Lingonberry	14	10	71.4	22	9	40.9	3.61	[0.71–20.35]	0.074
Tomato salad	42	37	88.1	40	29	72.5	2.81	[0.78–11.36]	0.075
Beef red wine	15	15	100.0	23	19	82.6	NA	NA	0.088
Pulled pork	20	20	100.0	21	19	90.5	NA	NA	0.157
Chips	13	0	0.0	24	3	12.5	0.00	[0.00–2.32]	0.184
Steak	13	0	0.0	24	3	12.5	0.00	[0.00–2.32]	0.184
Naan bread	36	28	77.8	23	15	65.2	1.87	[0.49–6.99]	0.290
Chickpea stew	27	9	33.3	24	11	45.8	0.59	[0.16–2.12]	0.361
Cabbage stew	14	9	64.3	22	11	50.0	1.80	[0.38–9.12]	0.400
Yogurt sauce	28	23	82.1	22	16	72.7	1.73	[0.36–8.42]	0.425
Cooked vegetables	16	13	81.3	20	14	70.0	1.86	[0.31–13.66]	0.439
Salmon mousse	20	15	75.0	20	13	65.0	1.62	[0.34–8.09]	0.490
Butter potato	15	13	86.7	23	18	78.3	1.81	[0.24–21.45]	0.514
Cucumber	23	21	91.3	38	36	94.7	0.58	[0.04–8.68]	0.600
Green beans	34	12	35.3	24	7	29.2	1.32	[0.38–4.87]	0.624
Rice	35	34	97.1	25	24	96.0	1.42	[0.02–114.42]	0.808
Spread	22	9	40.9	34	15	44.1	0.88	[0.26–2.95]	0.813
Bread	21	9	42.9	36	16	44.4	0.94	[0.27–3.16]	0.907
Tomato (fresh)	24	20	83.3	37	31	83.8	0.97	[0.20–5.27]	0.963
Cream	0	0	NA	2	2	100.0	NA		
Butter chicken	36	36	100.0	25	25	100.0	NA		

Exposure	Cases			Controls			Odds ratio		p value
	Total	Exposed	AR	Total	Exposed	AR	OR	95% CI	
Beetroot	0	0	NA	4	3	75.0	NA		
Sweet potato	0	0	NA	2	2	100.0	NA		
Vegetable stew	0	0	NA	2	2	100.0	NA		

AR: attack rate; CI: confidence interval; NA: not applicable; OR: odds ratio
